# Point-of-Care RNA-Based Diagnostic Device for COVID-19

**DOI:** 10.3390/diagnostics10030165

**Published:** 2020-03-18

**Authors:** Ting Yang, Yung-Chih Wang, Ching-Fen Shen, Chao-Min Cheng

**Affiliations:** 1 Institute of Biomedical Engineering, National Tsing Hua University, Hsinchu 300, Taiwan; tinayang0402@gmail.com; 2 Division of Infectious Diseases and Tropical Medicine, Department of Internal Medicine, Tri-Service General Hospital, National Defense Medical Center, Taipei 114, Taiwan; wystwyst@gmail.com; 3 Department of Pediatrics, National Cheng Kung University Hospital, College of Medicine, National Cheng Kung University, Tainan 704, Taiwan

At the end of 2019, the novel coronavirus disease (COVID-19), a fast-spreading respiratory disease caused by severe acute respiratory syndrome coronavirus 2 (SARS-CoV-2), was reported in Wuhan, China and has now affected over 123 countries globally. As of March 14, 2020, the death toll has exceeded 5400, and there have been 145,000 confirmed cases, causing not only a huge medical health burden but also tremendous economic losses worldwide [1]. Unlike previous coronaviruses that caused large-scale epidemics such as the Middle East Respiratory Syndrome (MERS) and Severe Acute Respiratory Syndrome (SARS), the transmission rate for COVID-19 is much higher, with an average of two to three people becoming infected for every already infected person [2]. Currently, there are two primary methods for diagnosing COVID-19: (1) a lateral flow immunoassay, which is a common point-of-care (POC) diagnostic approach that detects antibodies against specific viruses (e.g., SARS-CoV-2) in patient samples; and (2) a molecular-based assay. The current standard approach for screening COVID-19 requires a reverse real-time PCR assay (rRT-PCR), which can be carried out using a variety of clinical specimens, including bronchoalveolar lavage fluid, fibrobronchoscope brush biopsies, sputum, nasal swabs, pharyngeal swabs, feces, or blood [3]. This approach relies on expensive facilities, well-trained staff, and is often time-consuming, leaving a rapidly rising number of potential cases untested and opening a gaping hole in disease prevention efforts. Moreover, traveling to a clinical setting for testing increases the risk of spreading the disease and adds strain to a resource-limited healthcare system. For these reasons, an alternative, rapid, inexpensive, easy-to-use, and sensitive COVID-19 diagnostic tool must be developed for use by nonclinical individuals in their homes.

A recent study conducted by Prof. Jinzhao Song at the University of Pennsylvania in mid-February of 2020 describes a novel closed-tube COVID-19 assay that includes pathogen nuclei acid amplification and detection [4]. Because viral samples are small and standard practices require RT-PCR, false-negative test results are possible and patients may consequently become more seriously ill [5]. To improve COVID-19 diagnostic test sensitivity, Song’s group developed a closed-tube Penn-RAMP, a two-stage isothermal dsDNA amplification method that utilized both recombinase polymerase amplification (RPA) and loop-mediated isothermal amplification (LAMP) techniques in a single tube. To perform the LAMP test specifically for COVID-19, Song et al. selected conserved COVID-19 sequences using Clustal X and further designed LAMP primers. In order to make the detection process simpler, they used leucocrystal violet (LCV) dye as a chromogenic reagent, providing an obvious, deep violet color change that could be observed with the naked eye. The entire diagnostic process was relatively simple and required only a single tube for reaction. In this process, the RPA mixture was loaded onto the inside of the tube lid and the LAMP mixture (ratio of 1:9) was placed within the tube itself. The tube was subsequently sealed and incubated at 38 degrees Celsius for 15–20 min in a thermal cycler to facilitate the RPA reaction. The tube was then inverted several times and incubated at 63 degrees Celsius for 40 min. Because they did not have access to actual COVID-19 samples, Song et al. used samples of healthy nasal mucosa spiked with inactivated HIV particles and other pathogens as their test materials. The Penn-RAMP process provided greater sensitivity than RT-PCR or LAMP alone. When using limited viral load, Penn-RAMP provided 100 times better sensitivity than a single LAMP test. Compared to a LAMP assay, which requires sophisticated equipment and must be run at a fixed temperature, the Penn-RAMP process requires less energy cost, is easier to execute, and can be completed in clinical or home settings.

The abovementioned research describes essential materials and protocol for a single-tube COVID-19 test that can be completed without visiting a clinical setting. Because standard clinical RT-PCR testing is unlikely to meet rising test demand, there is a critical need for the development of alternative approaches for home-based point-of-care (POC) testing. These approaches may include LAMP assay and lateral flow assay techniques. Here, we describe a potential RNA-based POC diagnostic device for detecting COVID-19 that combines both a paper-based POC diagnostic device and LAMP assay technology. This convenient device can be integrated with a smartphone application to provide a rapid, sensitive, and highly accessible COVID-19 diagnostic tool (Figure 1). 

The concept for this tool is derived from previous research on paper-based nucleic acid detection employing RT-LAMP assay amplification [6]. Paper-based diagnostic tools have been widely applied for a variety of biochemical assays due to their low cost, ease of use, and speed. They have been employed to test a range of sample sources, including blood, urine, tears, and vaginal fluid, and could be easily adapted to accept nasal swab samples for viral detection. We have previously developed a paper-based platform for fluorescent nucleic acid screening using nucleic ladders. This research provided considerable insight into fluorescent probe selection and viral detection and required only a small sample volume. The potential rapid and easy-to-use paper-based LAMP assay for COVID-19 could be used in combination with a smartphone application to facilitate test results recording/sharing. Using this tool, a home-quarantined individual could easily self-collect a nasal swab sample; perform a LAMP assay; and observe a visible, colorimetric test result that could then be recorded and shared with clinicians or healthcare professionals via the internet. 

As the number of confirmed cases of COVID-19 rises rapidly throughout the world, more and more patients will require reliable testing. A rapid, inexpensive, and easy-to-use POC diagnostic device integrated with a smartphone could reduce transportation needs, lower the risk of spreading infection, alleviate the strain on the healthcare system, and mitigate the cost of care for both individuals and the government. We believe that research into the development of a paper-based RNA assay for use in combination with a smartphone application can provide new insights into designing POC COVID-19 diagnostics and ultimately improve the health care system to combat this and similar diseases. 

## Figures and Tables

**Figure 1:**
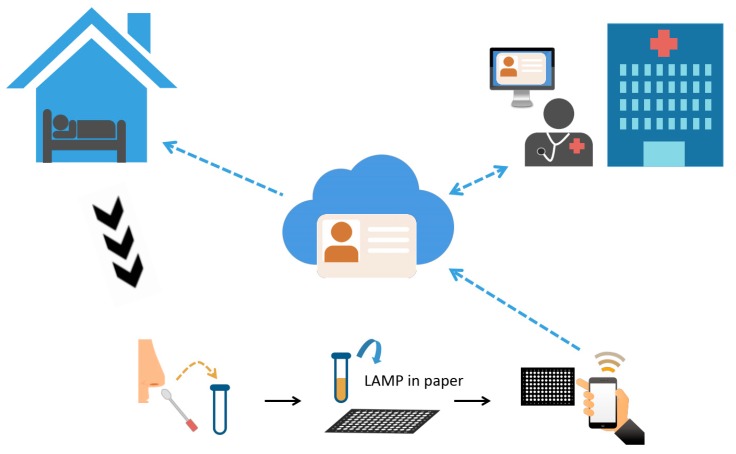
Schematic of the point-of-care (POC) RNA-based novel coronavirus disease (COVID-19) diagnostic device. In this figure, the conceptual workflow of integrating both point-of-care diagnostic and loop-mediated isothermal amplification (LAMP) assay with mobile device was presented. People at home quarantine can collect their infectious specimen through nasal swab; after adding specific reagents for LAMP reaction, the colorimetric result can be observed on paper. By recording the colorimetric change through a mobile phone camera, the user could upload the result to cloud storage by Internet. Clinicians or health-care specialist could thus analyze the result and offer immediate information for both patient and the government. Since the whole diagnostic process can be performed at home, the suspected case does not need to travel to hospital and can in turns lower the risk of spreading the disease.
